# External validation of Machine Learning models for COVID-19 detection based on Complete Blood Count

**DOI:** 10.1007/s13755-021-00167-3

**Published:** 2021-10-23

**Authors:** Andrea Campagner, Anna Carobene, Federico Cabitza

**Affiliations:** 1 DISCo, Università degli Studi di Milano-Bicocca, Milan, Italy; 2 Laboratory Medicine, IRCCS San Raffaele Scientific Institute, Milan, Italy

**Keywords:** COVID-19, Machine Learning, External validation, Calibration, Complete Blood count

## Abstract

**Purpose:**

The rRT-PCR for COVID-19 diagnosis is affected by long turnaround time, potential shortage of reagents, high false-negative rates and high costs. Routine hematochemical tests are a faster and less expensive alternative for diagnosis. Thus, Machine Learning (ML) has been applied to hematological parameters to develop diagnostic tools and help clinicians in promptly managing positive patients. However, few ML models have been externally validated, making their real-world applicability unclear.

**Methods:**

We externally validate 6 state-of-the-art diagnostic ML models, based on Complete Blood Count (CBC) and trained on a dataset encompassing 816 COVID-19 positive cases. The external validation was performed based on two datasets, collected at two different hospitals in northern Italy and encompassing 163 and 104 COVID-19 positive cases, in terms of both error rate and calibration.

**Results and Conclusion:**

We report an average AUC of 95% and average Brier score of 0.11, out-performing existing ML methods, and showing good cross-site transportability. The best performing model (SVM) reported an average AUC of 97.5% (Sensitivity: 87.5%, Specificity: 94%), comparable with the performance of RT-PCR, and was also the best calibrated. The validated models can be useful in the early identification of potential COVID-19 patients, due to the rapid availability of CBC exams, and in multiple test settings.

## Introduction

Since its initial spread in January 2020, the COVID-19 pandemic has so far affected more than 180 million people and caused more than 3 million deaths worldwide.

The reverse polymerase chain reaction (PCR) and the reverse transcriptase-PCR (rRT-PCR) are the gold standard tests for the detection of SARS-CoV-2 coronavirus, causative of COVID-19. However, both present known shortcomings such as long turnaround time, high costs, high false-negative rates (up to 15%) [12], the need for specialized equipment, and the associated shortage of reagents [13].

For these reasons, Machine Learning (ML) have been applied to hematological parameters [22, 27, 36] for a more rapid and cost-effective detection of the COVID-19 disease [13]. This is an interesting approach also in comparison to other alternative diagnostic methods, such as chest CT or X-rays. Indeed, although these latter methods have been associated with generally good performances [11, 18], most studies were found to be lacking in terms of methodological soundness [29]. Moreover, even if we assume the performance of those models can be replicated [3], they are also associated with much higher transaction costs than routine blood exams (including logistics and patient handling), and with lower safety, not only due to the high amount of radiation doses of CT procedures, but also to the risk of contamination of the radiology suites [16].

Although the potential of ML methods, based on hematochemical data, for COVID-19 detection is high, only a few models have been subjected to external validation [29].^1^ If we limit ourselves to ML models grounding on hematological data, among tens of publications, only the following publications report about an external validation procedure: [9, 26, 31, 35, 37]. Furthermore, to our knowledge only four studies studies are associated with either an online tool [5, 9, 19] or publicly available code [31] that interested healthcare practitioners could use on a set of their local cases (for which a definitive diagnosis of COVID-19 has been ascertained, possibly combining multiple techniques [33]) to perform what has been called *ecological* validation [8].

This lack of validation studies is quite striking in light of the need for fast and cost-effective diagnostic tests for COVID-19, and also in light of recent medical ML surveys [8, 36] and guidelines [17] which have strongly advocated the need to validate models *externally*. Indeed, lack of external validation has recently been noted in [29], together with lack of reproducibility [3, 36], as being one of the main challenges to the real-world adoption of ML-based approaches for COVID-19 diagnosis.

Furthermore, even when models are externally validated, they are very seldom validated also in terms of (probability) calibration. Though often neglected [10], calibration is a fundamental characteristics of clinical predictive models in that a calibrated model is capable to provide reliable probability estimates of the possible outcomes.^2^

For this reason, clinicians can use information about calibration to evaluate model’s trustworthiness [1], even more soundly than by relying on the model’s error rate (and other confusion-matrix metrics) as this latter can be affected by overfitting or data imbalance [30], to estimate pre-test probabilities, to undertake bayesian reasoning so as to rule-out conditions or prioritize interventions, and to combine results from different test techniques in multiple-testing settings so as to achieve much higher predictive values [2].

In order to address this gap in the literature, and to extend the work presented in [5, 9], in this contribution we present the validation process of 6 ML models that are based on the complete Blood Count (CBC) data originally collected at the Ospedale San Raffaele.^3^ To the purpose of the external validation, data were collected at two different hospitals, the hospital of Desio and the hospital of Bergamo, facilities of 383 and 1080 beds and 25 and 54 km away from the former setting, respectively. The above mentioned models were validated with respect to both error rate (through different metrics, including accuracy, sensitivity, specificity and AUC) and calibration. To this latter aim, other than the Brier score and the calibration plots, we also describe metrics that allow to understand the behavior of the models in regard to predictions associated with high probability scores, i.e the predictions on which the physicians would rely with higher confidence. Thus, the main objective of this study was to evaluate whether ML models for COVID-19 diagnosis, based on CBC data, could be robust to cross-site transportability and could thus be reliably deployed as medical decision support tools.

The rest of the article will be organized as follows. In “Methods” section we describe the validated models, focusing in particular on their training set and development procedures, as well as the external validation datasets. We also describe a set of metrics to evaluate the calibration of ML models. In Section 3 we report the results of the external validation study, while in “Discussion” section we discuss about the significance of the obtained results, as well as of validation studies more in general, we provide a comparison with existing state-of-the-art ML diagnostic models, and we illustrate possible uses of the validated models. Finally, in “Conclusion” section, we summarize our findings.

## Methods

**Fig. 1:**
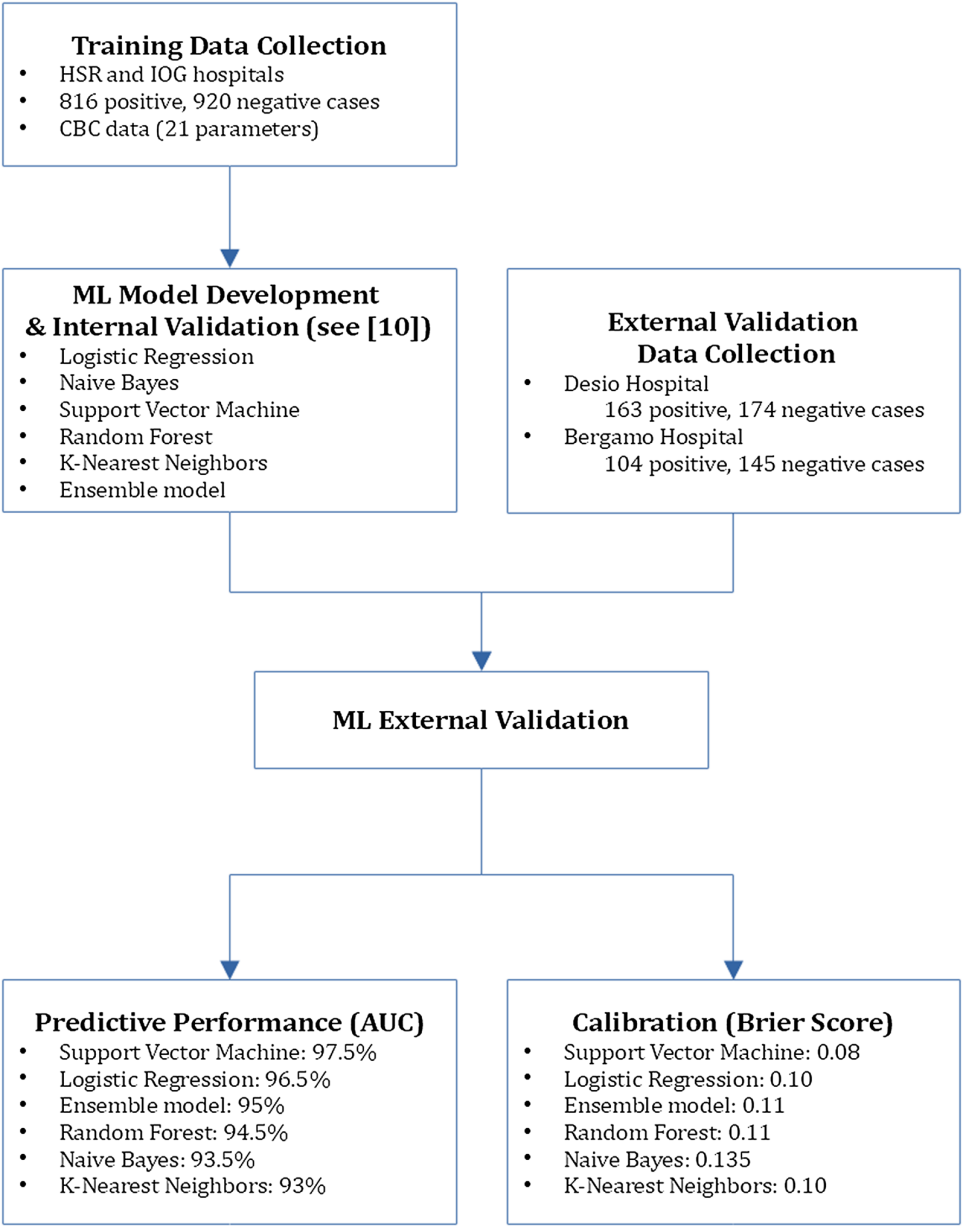
A summary illustration of the Methods and Results of the study. HSR denotes the data collected at IRCCS Hospital San Raffaele, IOG denotes the data collected at IRCCS Istituto Ortopedico Galeazzi, while CBC stands for Complete Blood Count

The study protocol (BIGDATA-COVID19) was approved by the Institutional Ethical Review Board (70/INT/2020) of IRCCS San Raffaele Scientific Institute in agreement with the World Medical Association Declaration of Helsinki. In this article, we adopt the MINIMAR [17] and IJMEDI [7] checklists for the reporting of ML models development and validation. A summary illustration of the Methods and Results of the study is reported in Fig. 1.

For the external validation, we considered 6 different ML models:

- Random Forest (maximum tree depth = 14, number of estimators = 419, robust feature scaling)
- Logistic Regression ($l_2$ regularization)
- SVM (RBF kernel, standard feature scaling)
- k-Nearest Neighbors (metric = euclidean, neighbors = 9, distance-based weights, Yeo-Johnson feature scaling)
- Naive Bayes (Yeo-Johnson feature scaling)
- A voting ensemble model, obtained as the (un-weighted) combination of the 5 previously mentioned models.

All training models were implemented in Python, using the scikit-learn [25] library (ver. 0.23.1), by means of a pipeline that encompassed: missing data imputation (using multivariate nearest neighbors-based imputation); feature scaling and feature selection (using recursive feature elimination [15]) steps; and hyper-parameter selection (using grid-search 5-fold nested cross-validation [32]).

The above mentioned ML models were trained on a set of 21 parameters, including the results of CBC exams, age (average: $60.9 \pm 0.9$ years), gender (57% male, 43% female) and the presence of COVID-19 related symptoms.

As previously explained in [9], the models were developed relying only on CBC data as these latter set of parameters can be acquired through rapid and inexpensive routine procedures. Furthermore, the wide availability of routine blood test, which can performed also in resource- or infrastructure-limited settings and countries, would make ML methods based only on these parameters more widely applicable (e.g., in third world countries).

The full set of parameters is shown in Table 1. The training dataset encompassed 816 COVID-19 positive and 920 negative cases, collected at the emergency departments (ED) of the IRCCS Hospital San Raffaele and the IRCCS Istituto Ortopedico Galeazzi of Milan (Italy). COVID-19 positivity was assessed by means of the rRT-PCR naso-pharyngeal swab. Uncertain cases were further assessed by means of either CT or X-ray examination. The training dataset was manually extracted from the electronic health record (EHR) of the two above mentione hospitals, and is available on Zenodo.^4^ We refer the reader to [9] for full details about model development and evaluation.

**Table 1:** The list of the 21 parameters, along with the target, used by the validated Machine Learning models

Parameter	Unit of measure	Train (missing)	Desio (missing)	Bergamo (missing)
Age	Years	60.93 ± 0.92 (3.11)	66.35 ± 1.97 (0.00)	54.38 ± 3.10 (0.00)
Hematocrit (HCT)	%	39.21 ± 0.26 (3.63)	38.20 ± 0.67 (0.00)	37.77 ± 0.91 (0.00)
Hemoglobin (HGB)	g/dL	13.14 ± 0.10 (3.63)	13.21 ± 0.25 (0.00)	12.86 ± 0.33 (0.00)
Mean Corpuscular Hemoglobin (MCH)	pg/Cell	29.21 ± 0.13 (3.63)	29.62 ± 0.34 (0.00)	30.41 ± 0.36 (0.00)
Mean Corpuscular Hemoglobin Concentration (MCHC)	g Hb/dL	33.45 ± 0.06 (3.63)	34.49 ± 0.16 (0.00)	33.98 ± 0.17 (0.00)
Mean Corpuscular Volume (MCV)	fL	87.29 ± 0.33 (3.63)	85.72 ± 0.86 (0.00)	89.44 ± 0.92 (0.00)
Red Blood Cells (RBC)	$10^12$/L	4.52 ± 0.03 (3.63)	4.49 ± 0.09 (0.00)	4.25 ± 0.11 (0.00)
White Blood Cells (WBC)	$10^9$/L	8.72 ± 0.22 (3.63)	9.81 ± 0.85 (0.00)	8.31 ± 0.88 (0.00)
Platelets (PLT1)	$10^9$/L	235.66 ± 4.43 (3.63)	220.23 ± 9.60 (0.00)	204.00 ± 14.10 (0.00)
Neutrophils (NE)	%	72.35 ± 0.62 (20.85)	75.03 ± 1.51 (0.00)	67.54 ± 2.13 (0.00)
Lymphocytes (LY)	%	18.58 ± 0.52 (20.85)	16.56 ± 1.24 (0.00)	21.90 ± 1.80 (0.00)
Monocytes (MO)	%	7.83 ± 0.18 (20.85)	7.17 ± 0.42 (0.00)	8.86 ± 0.58 (0.00)
Eosinophils (EO)	%	0.88 ± 0.08 (20.85)	0.74 ± 0.17 (0.00)	1.23 ± 0.26 (0.00)
Basophils (BA)	%	0.34 ± 0.01 (20.85)	0.18 ± 0.04 (0.00)	0.46 ± 0.05 (0.00)
Neutrophils (NET)	$10^9$/L	6.45 ± 0.21 (20.85)	7.47 ± 0.52 (0.00)	5.62 ± 0.53 (0.00)
Lymphocytes (LYT)	$10^9$/L	1.37 ± 0.04 (20.85)	1.63 ± 0.67 (0.00)	1.84 ± 0.60 (0.00)
Monocytes (MOT)	$10^9$/L	0.62 ± 0.03 (20.85)	0.64 ± 0.05 (0.00)	0.73 ± 0.11 (0.00)
Eosinophils (EOT)	$10^9$/L	0.07 ± 0.01 (20.85)	0.06 ± 0.01 (0.00)	0.09 ± 0.02 (0.00)
Basophils (BAT)	$10^9$/L	0.02 ± 0.00 (20.85)	0.02 ± 0.01 (0.00)	0.03 ± 0.00 (0.00)
COVID-19 specific symptoms at triage (suspect)	Yes/No	68%/32% (0%)	100%/0% (52%)	90%/10% (53%)
Gender	M/F	57%/43% (0%)	65%/35% (0%)	68%/32% (0%)
COVID-19 positivity (target)	Positive/Negative	53%/47%	52%/48%	58%/42%

For each continuous parameter and each dataset we report the mean and the extremes of the 95% confidence intervals, as well as the missing rate (in parenthesis). For the discrete features, as well as for the target, we report the distribution of values, as well as the missing rate (in parenthesis). The considered external validation sets had no missing values, except for the Suspect parameter

The average AUC of the ML models on the internal validation set, evaluated through nested 5-fold cross-validation^5^, was 0.85. Models were then retrained on the full set of training data, and have been made freely usable as a web-service.^6^

We validated the ML models on two different external datasets, separately: the Desio (DS, from the Desio Hospital) and the Bergamo dataset (BG, from the Bergamo Hospital). Both datasets encompass CBC data from COVID-19 positive patients retrospectively collected and assessed by means of rRT-PCR at the EDs in March/April, 2020 (163 and 104 subjects for Desio and Bergamo, respectively), and from true negative cases collected at the same EDs in 2019 (174 and 145 subjects for Desio and Bergamo, respectively). CBC analysis was performed by a Sysmex XN-9000 analyzer. The average age in the Desio and Bergamo datasets were, respectively $66.3 \pm 2.0$ and $54.4 \pm 3.1$ years. The distributions of biological sex were 65% males and 35% females, for the Desio dataset, and 68% males and 32% females, for the Bergamo dataset. Based on the proportion of COVID-19 positive cases in the two external validation datasets, and assuming a baseline AUC of 0.85, the two datasets were adequate in terms of sample size (minimum sample size equal to 234 and 239, for the Desio and Bergamo datasets respectively) [28].

**Fig. 2:**
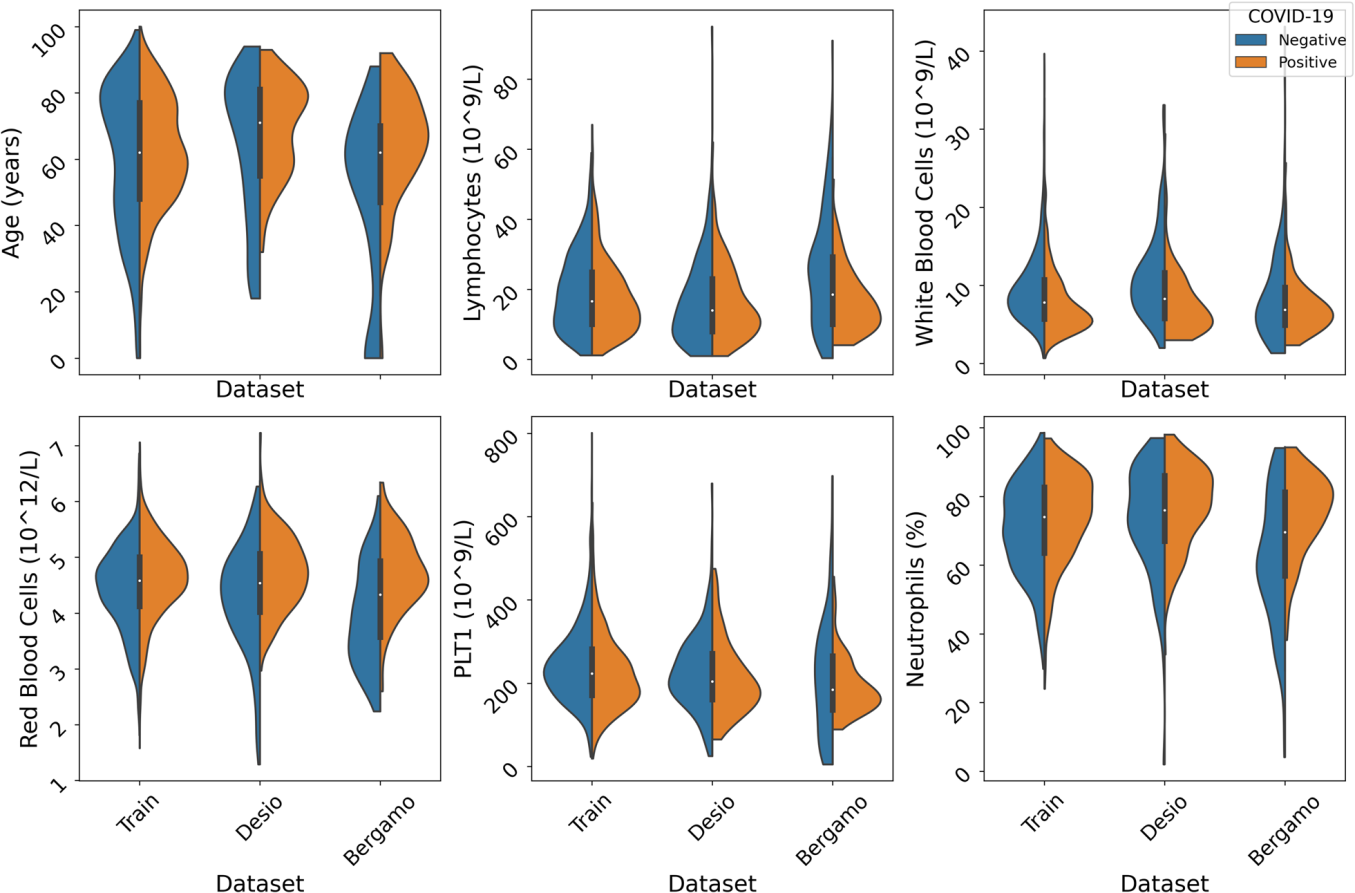
Violinplots of key CBC parameters in the training and validation datasets: White Blood Cells, Neutrophils, Lymphocytes, Red Blood Cells, platelets count and patient’s age (the parameters shown are the 6 most important features as reported in [9]). In the Suspect Symptoms Figure, NA means that the information was missing

**Fig. 3:**
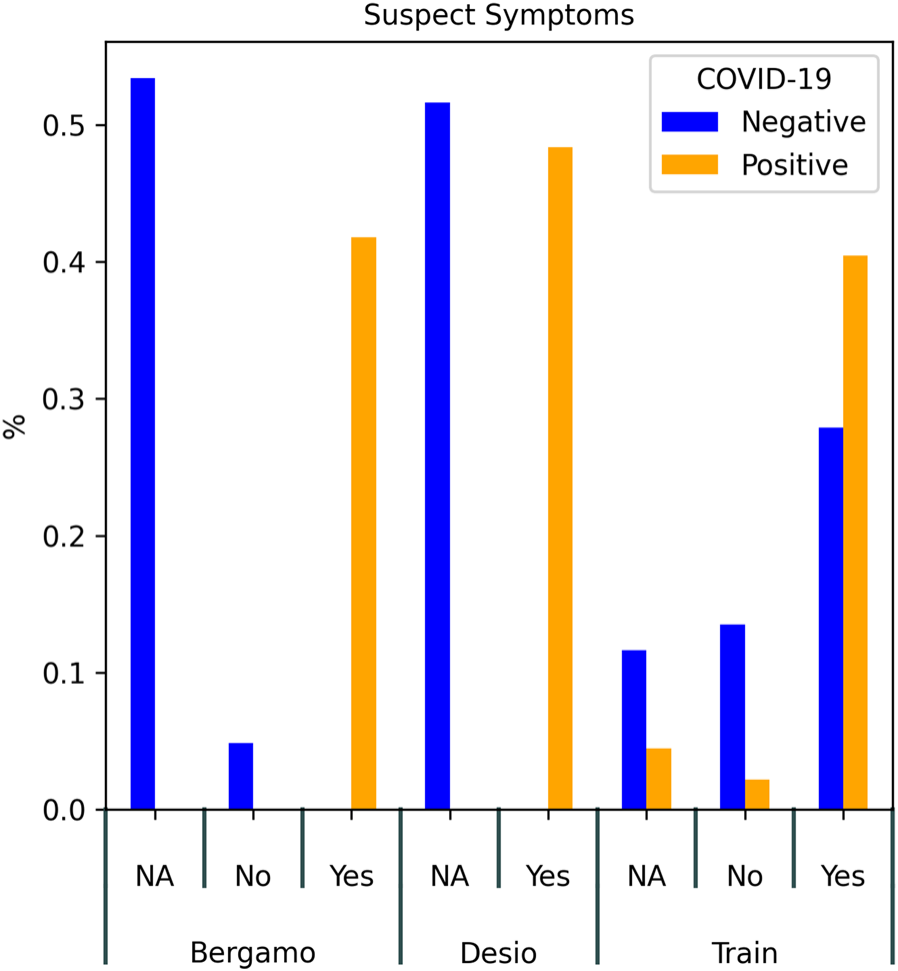
Boxplot of the distribution of presence of COVID-19 suspect symptoms. NA means that the information was missing

The external validation datasets were not affected by missing values, except for the Suspect feature (see Table 1). In this latter feature, the missing rates were 52% and 53%, for the Desio and Bergamo datasets, respectively. Distributions of key parameters in the training and validation datasets are reported in Figs. 2 and 3. The external validation was performed in terms of both error-based metrics (accuracy, sensitivity, specificity, false positive rate, false negative rate and AUC score), utility (in terms of Net Benefit), and calibration. With respect to calibration, in addition to the Brier score (which measures the deviations between probability scores on a quadratic scale), we describe an original set of metrics, whose goal is to better understand the performance of the models on the predictions they are most confident about (that is, so-called *highly-confident (HC) predictions*).

In this article we consider a threshold of 75% for defining HC predictions (for either the positive or negative class). We then report the values of standard metrics (accuracy, sensitivity, specificity, AUC) on this subset of instances, all together with the *Coverage*, i.e. the proportion of predictions for which the models were “highly confident”; as well as the *Total Variation* [20] on the HC predictions. This latter metric, in particular, is defined as follows:

1 $$\begin{aligned} \frac{1}{|Z|}\sum _{x_i \in Z} |y_i - h(x_i)| \end{aligned}$$

where $h(x_i)$ is the probability score, for the positive class, of model *h* on instance $x_i$; $y_i$ is the class associated with instance $x_i$; and $Z = \{ x_i : h(x_i) \ge 75\% \vee h(x_i) \le 25\% \}$ is the set of HC predictions.

## Results

**Fig. 4:**
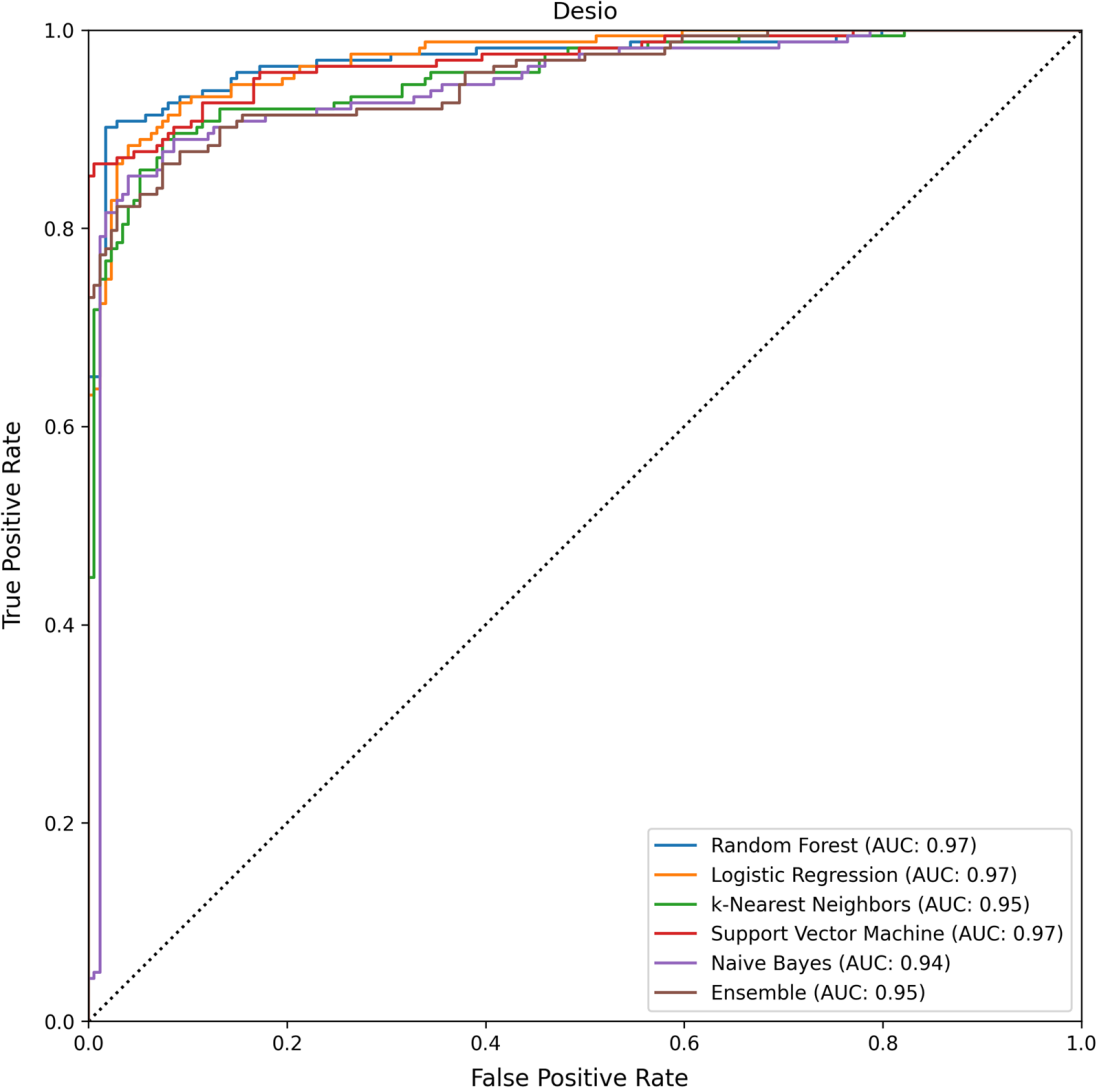
The results of the evaluated models on the Desio dataset. The performance of the models is reported in ROC space, along with the respective Area Under the ROC Curve (AUC)

**Fig. 5:**
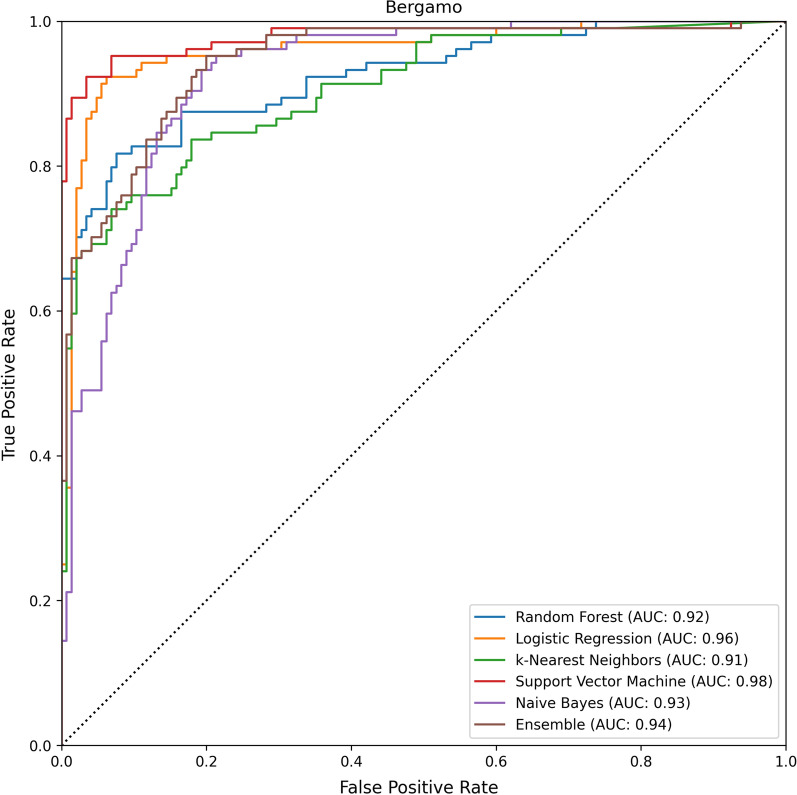
The results of the evaluated models on the Bergamo dataset

The average results, together with the results of the different models, are reported in Table 2. The ROC curves of the models and their respective AUCs, are reported in Figs. 4, 5.

**Fig. 6:**
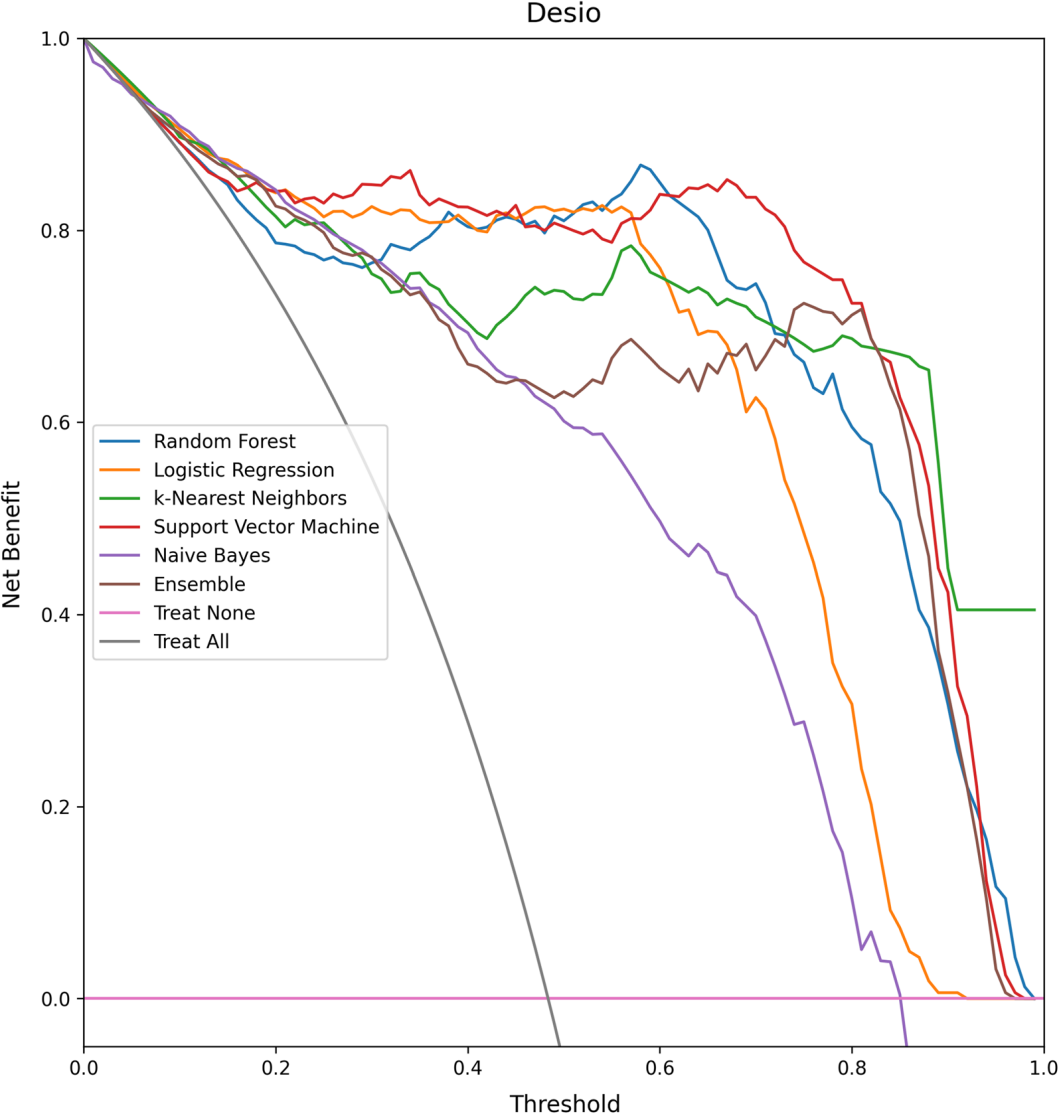
The decision curves of the evaluated models on the Desio dataset. The performance of the models is reported in Net Benefit space, showing the variation in Net Benefit with respect to the selection of a probability threshold. Treat None refers to the always negative (i.e. all patients considered as COVID-19 negative) baseline, while Treat All refers to the always positive (i.e. all patients considered as COVID-19 positive) baseline

**Fig. 7:**
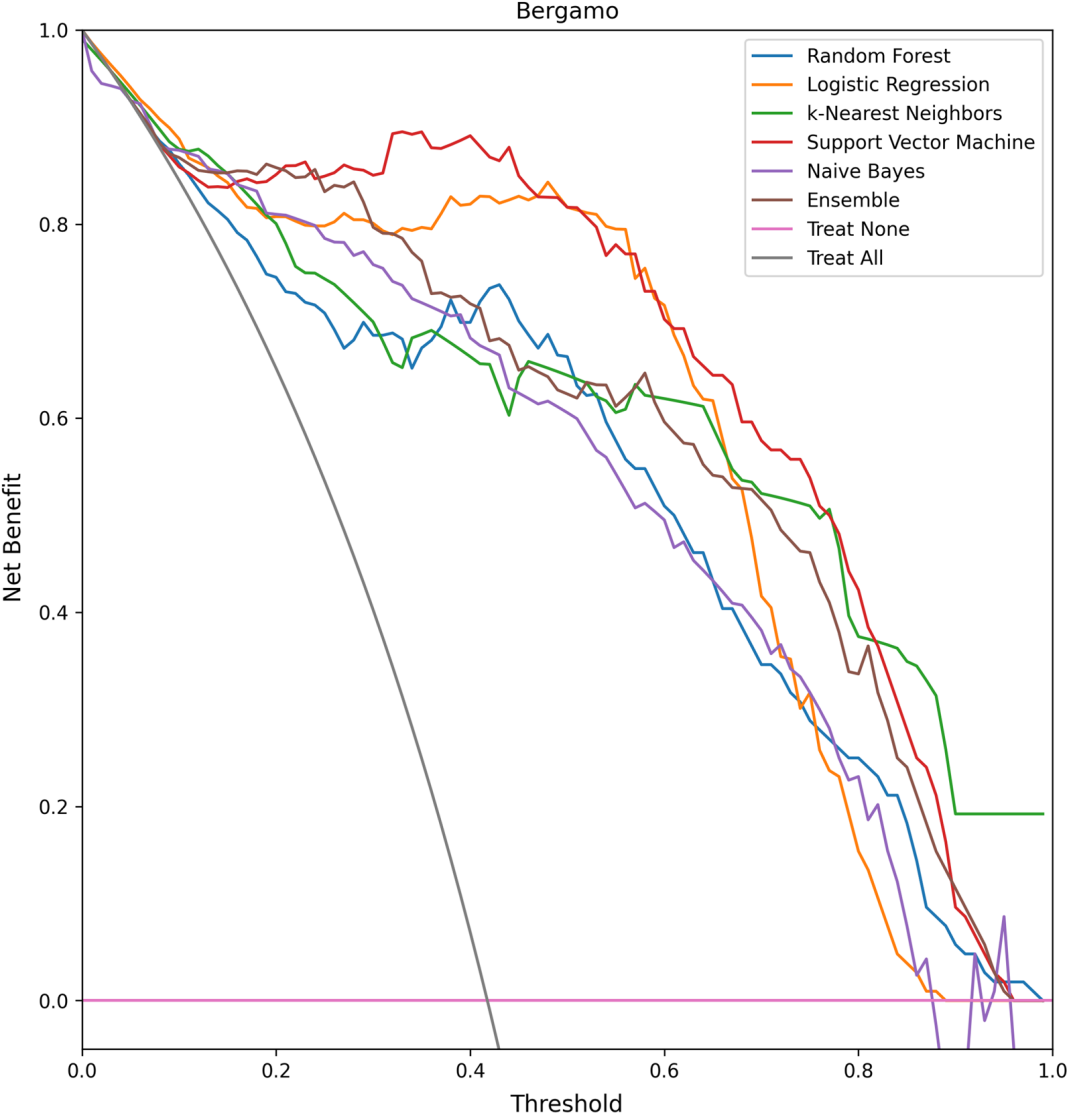
The decision curves of the evaluated models on the Bergamo dataset

On average, the AUC and accuracy of the models are, respectively, 95% and 87%. The Decision Curves of the models are reported in Figs. 6, 7. All models reported good predictive performance. In particular all models were consistently better than the Treat All baseline, while all models but Naive Bayes were consistently better than the Treat None baseline. The worse performing model (Naive Bayes) reported an average accuracy of 82.5% and an average AUC of 93.5%. The Naive Bayes model was also the worse calibrated one, with an average Brier score of 0.135, and the one with smallest Net Benefit (average 0.605). In particular, the Naive Bayes model reported a Net Benefit smaller than the Treat None baseline for all threshold values greater than 0.83. The overall best performing model, in terms of both AUC and Brier score, was Support Vector Machine with an average AUC of 97.5%, an average Brier score 0.08, and an average Net Benefit of 0.81. On average, the models reported better performances on the Desio dataset, in terms of Sensitivity, AUC, Net Benefit and Brier score. However, better Specificity was achieved on the Bergamo dataset. The models were not affected by gender bias. Indeed, the average accuracy on male patients was 86%, while on female patients was 89%. The difference was not significant (two-tailed Z score test, $z = -1.02, p=0.308$).

**Table 2:** The results of the evaluated models on the two external validation datasets: Desio dataset and Bergamo dataset

Model	Acc.	Sens.	Spec.	FPR	FNR	AUC	Brier	NB	Acc.	Sens.	Spec.	FPR	FNR	AUC	Brier	NB
	(DS) (%)	(DS) (%)	(DS) (%)	(DS) (%)	(DS) (%)	(DS) (%)	(DS)	(DS)	(BG) (%)	(BG) (%)	(BG) (%)	(BG) (%)	(BG) (%)	(BG) (%)	(BG)	(DS)
Random Forest	91	93	89	11	7	**97**	0.09	0.81	86	70	98	2	30	92	0.13	0.66
Logistic Regression	91	92	91	9	8	**97**	0.10	0.82	92	89	94	6	11	96	0.10	0.82
k-Nearest Neighbors	87	92	83	17	8	95	0.10	0.74	85	74	93	7	26	91	0.10	0.64
Support vector machine	90	91	89	11	9	**97**	0.08	0.79	93	84	99	1	16	**98**	0.08	0.83
Naive bayes	81	93	70	30	7	94	0.14	0.60	84	76	89	11	24	93	0.13	0.61
Ensemble	82	92	73	27	8	95	0.11	0.63	84	73	92	8	27	94	0.11	0.62
Average	87	92	82	18	8	96	0.10	0.73	87	78	94	6	22	94	0.11	0.70

Models were evaluated in terms of accuracy, sensitivity, specificity, area under the ROC curve (AUC), Brier score and Net Benefit. Values are reported in percent (%) format. For each dataset, the largest AUC values are highlighted in bold. We recall that, for the Brier score, the smaller the better

**Table 3:** The results of the evaluated models on the two external validation datasets: Desio dataset and Bergamo dataset

Model	HC	HC	HC	HC		Tot.	HC	HC	HC	HC		Tot.
	Acc.	Sens.	Spec.	AUC	Cov.	Var.	Acc.	Sens.	Spec.	AUC	Cov.	Var.
	(DS)	(DS)	(DS)	(DS)	(DS)	(DS)	(BG)	(BG)	(BG)	(BG)	(BG)	(BG)
Random Forest	0.98	0.98	0.97	0.99	0.55	0.15	0.97	0.91	1.00	0.97	0.39	0.17
Logistic Regression	0.99	0.98	1.00	1.00	0.50	0.16	0.97	0.92	0.99	0.97	0.53	0.16
k-Nearest Neighbors	0.96	0.98	0.93	0.98	0.69	0.12	0.93	0.87	0.98	0.95	0.65	0.15
Support Vector Machine	0.98	0.96	1.00	0.99	0.70	0.15	0.98	0.95	1.00	0.98	0.67	0.16
Naive Bayes	0.86	0.94	0.76	0.95	0.88	0.15	0.88	0.81	0.92	0.95	0.86	0.13
Ensemble	0.96	0.97	0.95	0.99	0.68	0.15	0.97	0.93	0.99	0.98	0.65	0.16
Average	0.95	0.97	0.93	0.98	0.67	0.15	0.95	0.90	0.98	0.97	0.62	0.15

Models were evaluated in terms of the HC metrics (i.e., metrics evaluated on instance on which the model reported a probability score greater than 75%, for either of the two classes): accuracy, sensitivity, specificity, area under the ROC curve (AUC). Coverage reports the proportion of HC predictions

**Fig. 8:**
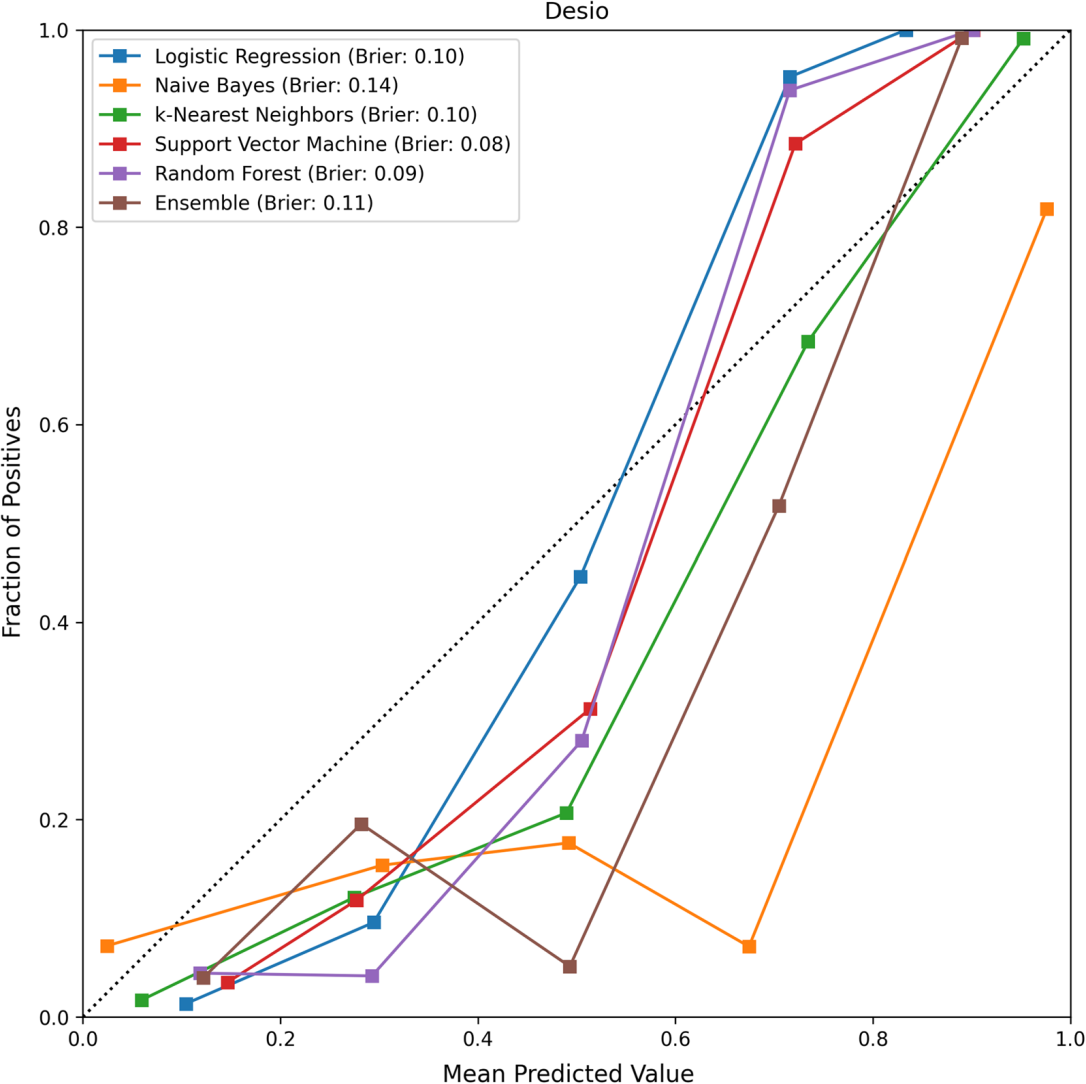
The calibration plot for the evaluated models on the Desio dataset, along with the respective Brier scores

**Fig. 9:**
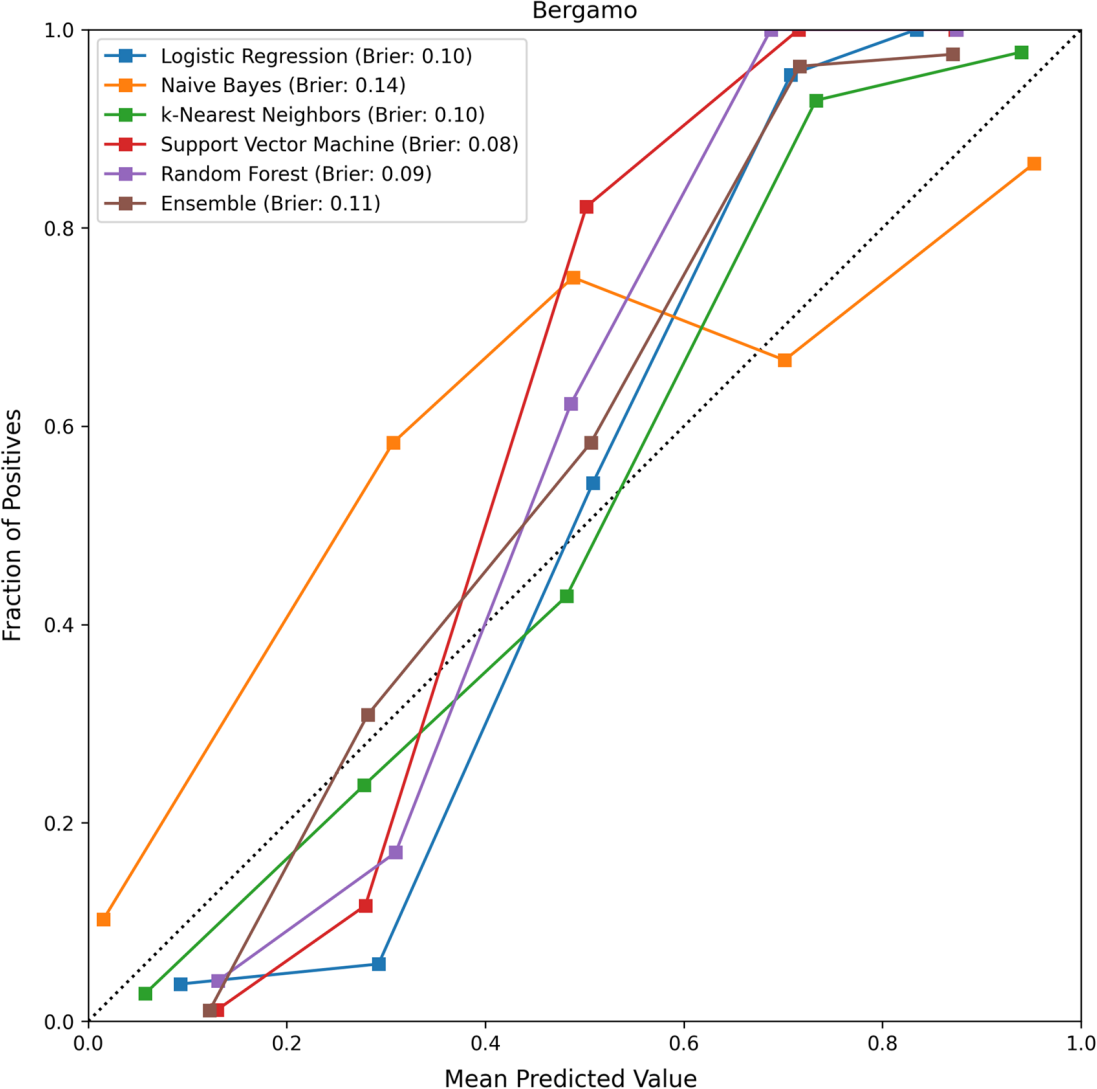
The calibration plot for the evaluated models on the Bergamo dataset, along with the respective Brier scores

The calibration (or reliability) plots for the evaluated models, and their respective Brier scores, are reported in Figs. 8, 9. The values of the HC metrics, Coverage and Total Variation are reported in Table 3. In all cases, the performance of the models on the Highly Confident instance improved compared to the results on all the instances: the average improvement in terms of AUC was 2.5%, while the average improvement in terms of accuracy was 8%. The best models in terms of both HC Accuracy and HC AUC were Logistic Regression and Support Vector Machine, both of which reported a value of 98% and 98.5%, respectively. These results suggest that the models were highly accurate on the instances less affected by epistemic uncertainty. In terms of Coverage, all models but Random Forest reported a Coverage greater than 50%. In particular, the best performing models (Logistic Regression and Support Vector Machine) reported an average coverage of 51.5% and 68.5%. All models reported a Total Variation greater than the corresponding Brier score: in particular, the best performing model in terms of Total Variation was k-Nearest Neighbors which reported an average value of 0.135.

**Fig. 10:**
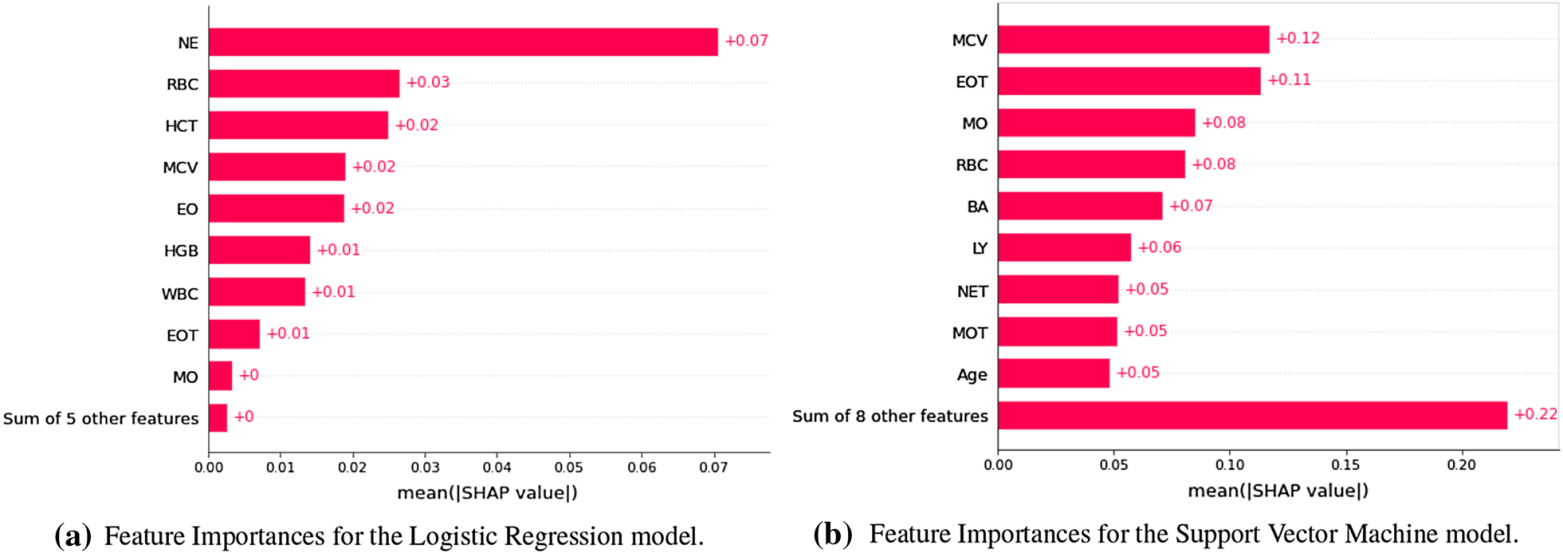
Feature importances for the Logistic Regression model. Feature importances for the Support Vector Machine model

The feature importances for the best performing models (namely, Logistic Regression and Support Vector Machine), computed on the external validation datasets using the Shapley values method [21], are reported in Fig. 10a, b. These two models used different features in their predictions. The Neutrophils percentage was found to be among the most predictive feature for the Logistic Regression model, while the most predictive feature for the SVM model was the Mean Corpuscolar Volume. Nonetheless, both models had a large degree of overlap in the features identified as most predictive (even more so, if we consider that each formula component was measured through two paired parameters). Indeed, Red Blood Cells and Mean Corpuscular Volume were among the 5 most predictive features for both models, and also different formula components (Neutrophils, Eosinophils and Monocytes) were found to be highly predictive. Notably, all these parameters have been previously recognized as highly predictive biomarkers for COVID-19 diagnosis [13, 38].

## Discussion

As reported above, all AUC scores are above 90% (see Figs. 4 and 5); moreover, the Brier scores are always lower than 0.15 (see Table 2), and the models exhibited excellent performance on the most confident predictions.

But what does this mean, practically speaking? A validated ML model that uses CBC data to detect COVID-19 can be adopted either as a complementary method to the RT-PCR test, for the fast and cost-effective identification of COVID-19 positive patients. Also other use cases are viable: even after the COVID-19 pandemics will have backed off to a more endemic and controlled disease, the fast triaging of admitted patients on the basis of CBC test results could facilitate healthcare practitioners in terms of prophylactic management and ward allocation. Furthermore, a validated CBC model can be useful for its probabilistic scores, as these can be used in multiple-test settings: to estimate Negative Predictive Values, so as to help general practitioners in ruling out COVID-19 positivity from subjects in self-quarantine; or to better estimate the prior probability of disease of other tests to detect COVID-19 and increase the reliability of their positive predictive value.

The models that we have validated compare favorably with the existing state of the art: more specifically, they outperform the model described by Yang et al. [37], which reported an AUC score of 84% and was, so far, the only ML model defined as having clinical viability [22]. Similarly, the reported results are competitive also with respect to the other works in the literature that have undergone external validation: Soltan et al. [31] report an AUC of 87%; Plante et al. [26] report an AUC of 91%, with high sensitivity (between 92.6% and 95.9%) but very low specificity (between 41.7%); Wu et al. [35] report an accuracy of 96% (sensitivity: 95%, specificity: 97%), though the model was described as being affected by bias [26, 36], both in terms of population size (the model was trained and externally validated on datasets encompassing only 146 and 74 patients, respectively) and task definition (the model was trained to distinguish COVID-19 patients from patients affected by other lung-related diseases, such as lung cancer or tuberculosis).

Compared to these other approaches [26, 31, 35, 37], the validated models were developed using more advanced pre-processing techniques, including multivariate imputation (as compared to e.g. median-based imputation in [31, 37]) and extensive hyper-parameter optimization [9]. Furthermore, as described in [9], the gold standard used for training the validated models was obtained by means of a composite test which, for the more uncertain cases, combined the result of the molecular swab with the result of chest radiography and/or chest X-ray, so as to minimize labeling uncertainty, improve over the sensitivity of the molecular swab alone [34], and thus improve the data quality. Finally, differently from the approaches described in [26, 31, 35, 37], the models we developed to detect COVID-19 are based on demographic and CBC parameters only. As mentioned in the introduction, this is a fast and inexpensive diagnostic test, which is also less subject to analytic and biological variability as compared to other biomarkers [6].

Interestingly, the performance of the validated models was comparable with the performance of other, non ML-based diagnostic tests. Indeed, as highlighted in a recent systemic review [4], the average specificity of the best performing model (i.e. Support Vector Machine) was higher than all other reviewed diagnostic tests except for blood-based IgG immunological tests, while its sensitivity was higher than all other reviewed diagnostic tests except for sputum-based RT-PCR and Computed Tomography [4]. The proposed ML approaches, therefore, offer a good trade-off between sensitivity and specificity, with performance (in terms of AUC) comparable to that of the RT-PCR. Being based on routine blood tests, i.e. a rapidly available and inexpensive testing methodology, the validated ML models could be useful in the rapid identification and triaging of COVID-19 infections, as well as in multiple test settings, in combination with the gold standard RT-PCR test or other diagnostic approaches, so as to improve sensitivity and specificity.

Our models also report good calibration. Indeed, the best performing model (Support Vector Machine) reported a Brier score of 0.08. In order to better understand the reliability and calibration of the validated models’ probability scores, we can observe the values for the HC metrics in Table 3. All performance metrics, of all models, improved when we consider the instances where the models achieved high confidence in the prediction: all measures are above 95%. This means that most of the instances that had been wrongly classified were associated with greater model uncertainty (hence, lower probability scores). In particular, the most accurate model (that is, the Support Vector Machine model) reports an HC specificity equal to 1. This means that all “highly confident” predictions on negative instances were correct, thus proving that our models can be an effective tool for ruling-out a COVID-19 diagnosis.

Furthermore, all models report coverage higher than 50% and small Total Variation. In regard to coverage, the above result means that at least one half of the predictions were produced with high confidence and hence could be practically useful to physicians^7^. In regard to the total variation,we recall that a model associating all positive instances with a probability score of at least 75% (and all negative instances with a probability no greater than 25%) would result in a Total Variation value $\le$ 0.25. Thus, a model which reports a Total Variation lower than 25%, as the validated models described in this article, makes few error on its HC predictions and its probability scores on the HC predictions are well-calibrated.

## Conclusion

In this article, we reported about the external validation of 6 state-of-the-art ML models for COVID-19 diagnosis based on routine hematochemical parameters. The ML models reported excellent performance on two different, independent, external validation sets, both in terms of diagnostic accuracy and calibration. In particular, the best performing model (Support Vector Machine) reported an average AUC of 97.5% (Sensitivity: 87.5%, Specificity: 94%), out-performing the existing state-of-the-art ML methods, and reaching a performance comparable with the gold standard diagnostic tests (i.e. RT-PCR). Thus, being based on routine, rapidly available and inexpensive blood tests, the validated methods could be useful for the early identification of COVID-19 infection, due to the rapid availability of CBC exams as compared to RT-PCR, as well as in multiple test settings, in combination with other diagnostic tests, so as to improve sensitivity and specificity, or to provide prior probabilities for Bayesian reasoning. Following the recommendations reported in [22], the data used for model development has been made publicly available (on Zenodo^8^), so that authors of other studies amd developers of other ML tools for COVID-19 detection could use those data to perform external validations of their models.

Moreover, the models that we have validated in this paper have been made freely available online as a web tool^9^. For this reason, they could be easily adopted in developing countries as well as in any country facing a rapid increase in contagions, since CBC is a widely adopted diagnostic investigation [24]. Moreover, this web tool, which so far has been used more than 1300 times, has been designed to visually show prediction results in terms of probability scores, so as to be more interpretable and informative to both specialists and lay people [22].

## Data Availability

The training dataset is freely available on Zenodo. URL: https://zenodo.org/record/4081318#.YAFe5xYo-Uk The validation datasets will be made available on the Zenodo platform after publication.

## Footnotes

1 Practically speaking, to *externally validate* [23] a ML model means to evaluate the model’s performance on data that have been collected in settings different from the ones involved in the training and test of the model.

2 Intuitively speaking, if a calibrated model yields a probability score of 80% on a particular instance (and hence prediction), it means that *on previously seen cases* with the same prediction and probability score, the model has made the right prediction 8 times out of 10; this means that calibration assessment provides an idea of *case-wise accuracy*, while traditional measures only account for an aggregated (average) notion of accuracy.

3 The Ospedale San Raffaele is a 1350-beds and 3400-employee teaching hospital in Milan, Italy, i.e., one of the most affected regions in the world during the first wave of the disease [14]

4 https://zenodo.org/record/4081318#.YAFe5xYo-Uk

5 Thus, during internal validation, each model was trained with 64% of the data; hyper-parameter optimization was performed with 16% of the data; and evaluation on the remaining 20% of data

6 https://covid-19-blood-ml.herokuapp.com/

7 That said, discussing whether physicians should rely also on low-confidence predictions is beyond the scope of this contribution

8 https://zenodo.org/record/4081318#.X46whND7TIU

9 https://covid-19-blood-ml.herokuapp.com/

## Code availability

The developed models have been made freely usable as a web-service. URL: https://covid-19-blood-ml.herokuapp.com/.
